# Seasonal variation of vasopressin and its relevance for the winter peak of cardiometabolic disease: A pooled analysis of five cohorts

**DOI:** 10.1111/joim.13489

**Published:** 2022-04-17

**Authors:** Sofia Enhörning, Olle Melander, Gunnar Engström, Sölve Elmståhl, Lars Lind, Peter M. Nilsson, Mats Pihlsgård, Simon Timpka

**Affiliations:** 1 Perinatal and Cardiovascular Epidemiology, Department of Clinical Sciences in Malmö, Lund University, Malmö, Sweden; 2 Department of Internal Medicine, Skåne University Hospital, Malmö, Sweden; 3 Department of Clinical Sciences in Malmö, Lund University, Malmö, Sweden; 4 Department of Clinical Sciences in Malmö, Division of Geriatric Medicine, Lund University, Malmö, Sweden; 5 Department of Medical Sciences, Uppsala University, Uppsala, Sweden; 6 Internal Medicine—Epidemiology, Department of Clinical Sciences Malmö, Lund University, Malmö, Sweden; 7 Department of Obstetrics and Gynecology, Skåne University Hospital, Malmö, Sweden

**Keywords:** body mass index, copeptin, coronary artery disease, diabetes mellitus, seasonality

## Abstract

**Background:**

Vasopressin concentration is typically higher at night, during stress, and in males, but readily lowered by water intake. Vasopressin is also a causal candidate for cardiometabolic disease, which shows seasonal variation.

**Objective:**

To study whether vasopressin concentration varies by season in a temperate climate.

**Methods:**

The vasopressin surrogate marker copeptin was analyzed in fasting plasma samples from five population-based cohorts in Malmö, Sweden (*n* = 25,907, 50.4% women, age 18−86 years). We investigated seasonal variation of copeptin concentration and adjusted for confounders in sinusoidal models.

**Results:**

The predicted median copeptin level was 5.81 pmol/L (7.18 pmol/L for men and 4.44 pmol/L for women). Copeptin exhibited a distinct seasonal pattern with a peak in winter (mid-February to mid-March) and nadir in late summer (mid-August to mid-September). The adjusted absolute seasonal variation in median copeptin was 0.62 pmol/L (95% confidence interval [CI] 0.50; 0.74, 0.98 pmol/L [95% CI 0.73; 1.23] for men and 0.46 pmol/L [95% CI 0.33; 0.59] for women). The adjusted relative seasonal variation in mean log copeptin z-score was 0.20 (95% CI 0.17; 0.24, 0.18 [95% CI 0.14; 0.23] in men and 0.24 [95% CI 0.19; 0.29] in women). The observed seasonal variation of copeptin corresponded to a risk increase of 4% for incident diabetes mellitus and 2% for incident coronary artery disease.

**Conclusion:**

The seasonal variation of the vaso-pressin marker copeptin corresponds to increased disease risk and mirrors the known variation in cardiometabolic status across the year. Moderately increased water intake might mitigate the winter peak of cardiometabolic disease.

## Introduction

Several vasoactive and metabolic hormones exhibit seasonal variation [1–3]. Furthermore, incident coronary artery disease (CAD) [4, 5], insulin secretion and resistance [6, 7], and levels of many cardiometabolic risk factors [8, 9] increase in winter. Seasonal variation of temperature, infections, physical activity, and dietary patterns have been proposed to explain the higher incidence of CAD and worse metabolic profile during the cold season, but the underlying mechanisms remain unknown.

The seasonal variation of vasopressin (VP), the key hormone in regulating water balance [10], has not been studied at the population level. According to the current physiological paradigm, VP concentration in plasma increases with stress [11], at night [12], and with higher ambient temperature [13] to reduce renal water excretion. VP concentration can now be estimated via the surrogate marker copeptin [14], which is stable in vitro and therefore has facilitated epidemiological and experimental studies identifying VP as a causal candidate for cardiometabolic disease development [10, 15–17]. Both VP and copeptin secretion is readily lowered by increased water intake [16, 17]. Clinically, copeptin is relevant as a marker of VP secretion in the hypertonic saline infusion test for the diagnosis of central diabetes insipidus, that is, low VP secretion [18].

To study the seasonal pattern of VP concentration in adults, we have utilized individual-level data on copeptin analyzed in fasting plasma samples from five population-based cohorts collected during three decades in the city of Malmö, Sweden. The aim of the study was to investigate the extent to which VP concentration varies across the calendar year in a temperate climate.

## Materials and methods

### Study sample

We have combined data from five population-based observational cohort studies (total *n* = 25,907, age 18−86 years, 50.4% women) conducted in Malmö, Sweden, during 1992−2018, including (I) the Malmö Diet and Cancer—Cardiovascular Cohort (MDC-CC, *n* = 5028, age 46−68 years, 59.4% women, plasma samples collected during 1992−1994), (II) the Malmö Preventive Project (MPP, *n* = 5338, age 57−86 years, 30.2% women, samples collected during 2002−2006), (III) the EpiHealth Malmö Cohort (*n* = 8013, age 45−76 years, 55.7% women, samples collected during 2012−2017), (IV) the Malmö Offspring Study (MOS, *n* = 2080, age 18−71 years, 52.5% women, samples collected during 2013−2018), and (V) the Swedish CArdioPulmonary BioImage Study Malmö Cohort (SCAPIS, *n* = 5448, age 50−65 years, 53.2% women, samples collected during 2014−2018). The analysis set included all participants that had complete data on age, sex, copeptin, body mass index (BMI), physical activity, and blood sampling date. Detailed information about each cohort is included in the online Supplement.

All studies were approved by the local ethical committees. Written informed consent was obtained from each participant. The pooled analysis was approved by the Swedish Ethical Review Authority (Dnr 2020−04422).

### Analyses of VP (copeptin) concentration

In all five studies, VP concentration was estimated by using the VP marker copeptin [19]. Fasting plasma samples were sampled in EDTA plasma tubes and stored at −80°C until analysis. Samples were drawn in all studies after an overnight fast, except in the EpiHealth cohort, in which 6 h of fasting was required. The Brahms CT-proAVP LIA assay was used in the MDC-CC and MPP cohorts and the Brahms Copeptin proAVP KRYPTOR assay was used in the EpiHealth, SCAPIS, and MOS cohorts. The two analytical methods have been shown to have a good correlation [20].

### Data collection of covariables

The age of participants was collected at recruitment and analyzed as age in years. Data on smoking status (yes or no) and leisure time physical activity were collected through self-administered questionnaires. Participants' weight and height were registered by study staff on site in all five cohorts, and we calculated BMI as kg/m^2^ before analyses. Diabetes mellitus (diabetes) status and CAD (prevalent and incident) was ascertained by several different national and regional registers in the MDC-CC, the MPP, and the EpiHealth cohorts as detailed in the online Supplement.

### Geographical location and climate

The city of Malmö (population 350,000) is located by the sea in southernmost Sweden. The climate is temperate with the mean temperature in the coldest months (January and February) being approximately 0°C and in the warmest months (July and August) being approximately 20°C. The length of daylight varies across the year with a peak of 16 h in June and a nadir of less than 8 h in December (Fig. S1).

### Statistical analyses

Before plotting the pooled copeptin data descriptively by month, we log transformed copeptin and further calculated z-scores by cohort and sex, as the copeptin data are highly skewed, the analysis differ by cohort, and men have higher plasma concentration of copeptin [21].

Based on the visualized descriptive pattern of seasonal variation in log copeptin z-score, we utilized sinusoidal regression to model the seasonal variation of copeptin and adjust for confounders. To facilitate interpretation, we complementarily used linear mean and median regression for analysis. Analyzing log copeptin z-scores with standard mean regression allowed us to analyze the relative seasonal variation and to directly compare the descriptive (crude) data with estimates from a confounder-adjusted model. In order to also present absolute seasonal variation on a scale relevant for copeptin (pmol/L), we complementarily analyzed untransformed copeptin using confounder-adjusted median regression. In the confounder-adjusted models, we included variables that are both linked to copeptin concentration and that potentially varied by season in the pooled analysis (e.g., BMI, physical activity, cohort). The sinusoidal pattern of the seasonal variation of copeptin was reflected by incorporating cosine and sine terms in each model. From these mean and median sinusoidal regression models, we concomitantly estimated (I) the magnitude of difference between the peak and nadir of copeptin across the calendar year (i.e., 2× the sinusoidal amplitude) and (II) the timing of the peak and nadir in copeptin across the calendar year (i.e., the curve's phase shift). To report the level of median copeptin around which the seasonal variation occurred, we estimated predicted median copeptin by weighting covariates to reflect the pooled cohort data and letting the sine and cosine terms be zero. Analyses were performed with interaction terms by sex, to allow for separate estimates of sinusoidal curves, and we also performed analyses on interaction by age (≤60 years vs. >60 years of age) and BMI (BMI <25 kg/m^2^ vs. BMI ≥25 kg/m^2^).

To relate the magnitude of seasonal variation in copeptin to incident cardiometabolic disease, we utilized the subset of three cohorts (MDC, MPP, and EpiHealth) with available data on prevalent and incident diabetes and CAD. Separately for each of these two outcomes, we first studied the association between mean log copeptin z-score and incident disease among initially disease-free individuals by using multivariate adjusted (cohort, age, BMI, smoking status, physical activity) proportional hazard models. We then utilized the model estimates to calculate the risk of incident disease corresponding to the observed seasonal variation in mean copeptin for both diabetes and CAD.

We also performed a set of additional analysis. First, we analyzed the extent to which the seasonal variation of copeptin was affected by creatinine levels. For this analysis, we utilized the four cohorts (MDC-CC, MPP, MOS, and SCAPIS) with available data on plasma creatinine (excluding *n* = 303 with missing data on creatinine). Second, we analyzed the extent to which the seasonal variation of copeptin was affected by smoking status. For this analysis, we excluded participants (*n* = 3885) without available data on smoking status. Third, to analyze the seasonal variation of copeptin in participants without cardiometabolic disease, we repeated the main analysis in the three cohorts with incident disease data but first excluded all participants with prevalent diabetes or CAD (total exclusions *n* = 10,323, including MOS and SCAPIS).

All analyses are reported with 95% confidence intervals (CIs). Statistical analyses were performed by using the statistical software SAS 9.4, Cary N.C.

## Results

The pooled baseline characteristics of included participants from all five cohorts, including plasma copeptin concentrations, are displayed in Table 1. Corresponding characteristics presented by cohort can be found in Table S1.

**Fig. 1:**
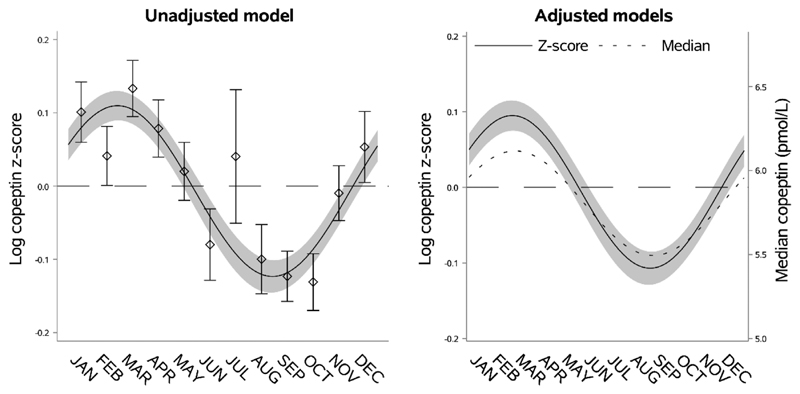
Seasonal variation of vasopressin estimated through copeptin. Left panel: seasonal variation of copeptin presented as mean log copeptin z-score (standardized by cohort and sex) by month with 95% confidence intervals. The solid line shows an unadjusted sinusoidal regression model fit to the data. Right panel: seasonal variation of copeptin presented as adjusted mean log copeptin z-score by month (solid line) with 95% confidence intervals (grey area). The dotted line shows seasonal variation of copeptin as adjusted median copeptin. Plotting of models is based on average age and body mass index and proportions of categories of sex, physical activity, and cohort.

VP concentration, estimated through copeptin, exhibited marked seasonal variation with the highest level found from mid-February to mid-March and the lowest level from mid-August to mid-September (Fig. 1, Table S2). The relative seasonal variation in log copeptin z-score in the adjusted model (0.20 [95% CI 0.17; 0.24]) was comparable to the plotted unadjusted data. In the adjusted median regression analysis, the seasonal variation exhibited a similar pattern (Fig. 1) and the absolute difference in copeptin was 0.62 pmol/L (95% CI 0.50; 0.74) between the seasonal peak compared to the nadir (Table S2). The seasonal variation of copeptin was also visible when the five cohorts were analyzed separately (Figs S2−S6).

### Seasonal variation of copeptin by sex

**Fig. 2:**
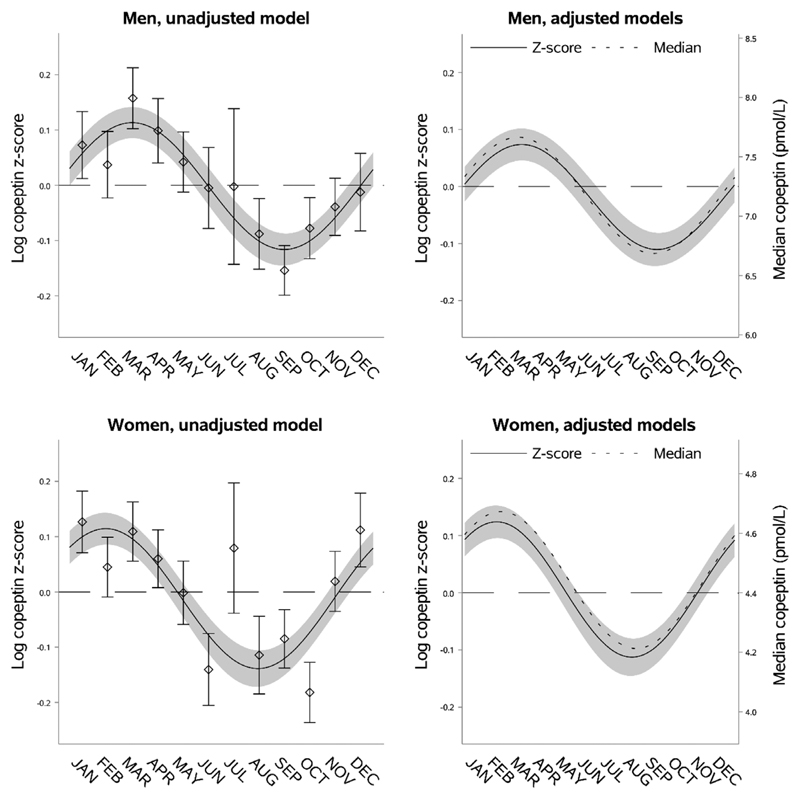
Seasonal variation of vasopressin estimated through copeptin by sex. Left upper panel: seasonal variation of copeptin in men presented as mean log copeptin z-score (standardized by cohort and sex) by month with 95% confidence intervals. The solid line shows an unadjusted sinusoidal regression model fit to the data. Right upper panel: seasonal variation of copeptin in men presented as adjusted mean log copeptin z-score by month (solid line) with 95% confidence intervals (grey area). The dotted line shows seasonal variation of copeptin as adjusted median copeptin. Plotting of models is based on average age and body mass index and proportions of categories of physical activity and cohort. Left lower panel: seasonal variation of copeptin in women similarly presented as that for men in the left upper panel. Right lower panel: seasonal variation of copeptin in women similarly presented as that for men in the right upper panel.

As expected, men had higher predicted median copeptin (7.18 pmol/L [95% CI 7.10; 7.26]) compared to women (4.44 pmol/L [95% CI 4.39; 4.50]) and men also had a higher absolute seasonal variation (0.98 pmol/L [95% CI 0.73; 1.23]) than women (0.46 pmol/L [95% CI 0.33; 0.59]). However, there was no significant difference in relative seasonal variation (0.18 [95% CI 0.14; 0.23] in men vs. 0.24 [95% CI 0.19; 0.29] in women, difference −0.053 [95% CI −0.12; 0.015]). Results from the mean regression modelling suggested a 1-month (34 days [95% CI 13; 54]) earlier peak and nadir of copeptin in women compared to men, and this was supported by the corresponding median regression analysis (Fig. 2, Table S3).

### Seasonal variation of copeptin by age

**Fig. 3:**
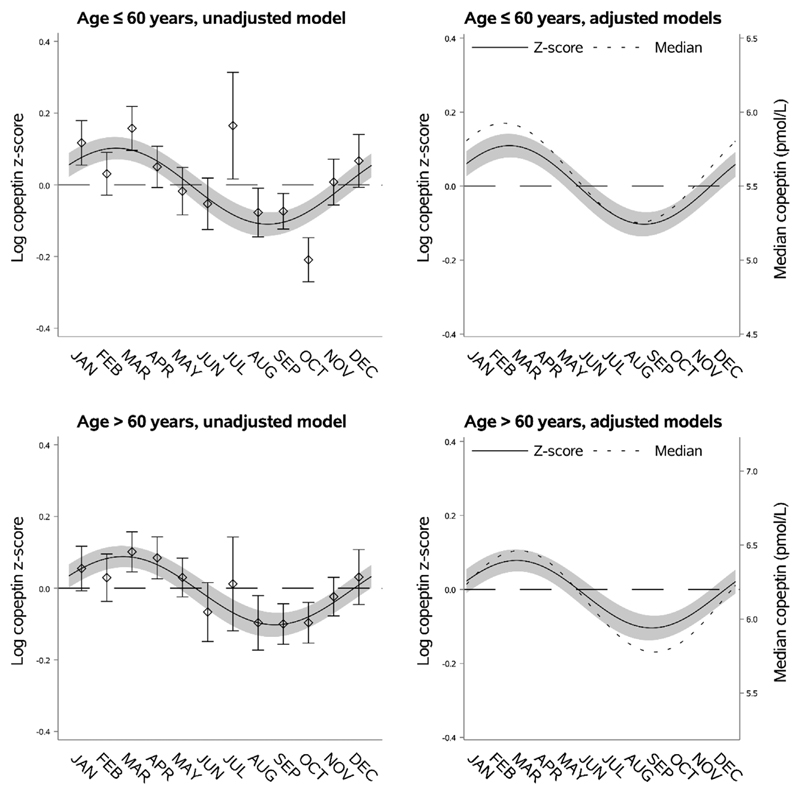
Seasonal variation of vasopressin estimated through copeptin by age. Left upper panel: seasonal variation of copeptin in individuals aged ≤60 years presented as mean log copeptin z-score (standardized by cohort and sex) by month with 95% confidence intervals. The solid line shows an unadjusted sinusoidal regression model fit to the data. Right upper panel: seasonal variation of copeptin in individuals aged ≤60 years presented as adjusted mean log copeptin z-score by month (solid line) with 95% confidence intervals (grey area). The dotted line shows seasonal variation of copeptin as adjusted median copeptin. Plotting of models is based on average body mass index and proportions of categories of sex, physical activity, and cohort. Left lower panel: seasonal variation of copeptin in individuals aged >60 years similarly presented as that for those with age ≤60 years in the left upper panel. Right lower panel: seasonal variation of copeptin in individuals aged >60 years similarly presented as that for those with age ≤60 years in the right upper panel.

Individuals ≤60 years of age had lower predicted median copeptin (5.59 pmol/L [95% CI 5.52; 5.66]) compared to individuals aged >60 years (6.12 pmol/L [95% CI 6.05; 6.19]). However, we found no difference in relative or absolute seasonal variation of copeptin (Fig. 3, Table S4). There was a tendency of earlier peak and nadir in individuals <60 years compared to those aged >60 years (difference 21 days, 95% CI −3; 45) (Fig. 3, Table S4).

### Seasonal variation of copeptin by BMI

**Fig. 4:**
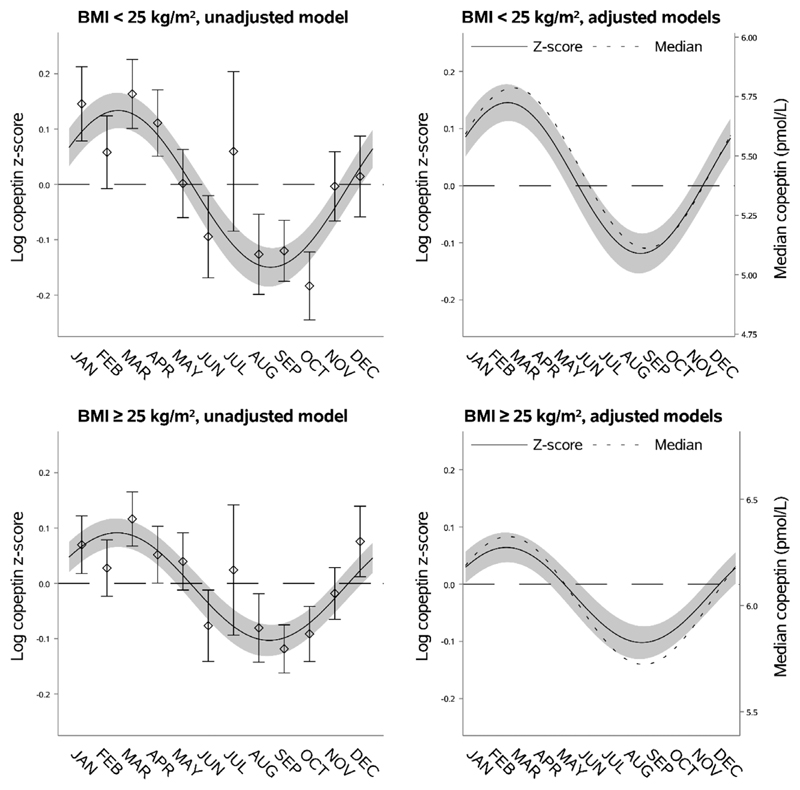
Seasonal variation of vasopressin estimated through copeptin by body mass index (BMI). Left upper panel: seasonal variation of copeptin in individuals with BMI <25 kg/m^2^ presented as mean log copeptin z-score (standardized by cohort and sex) by month with 95% confidence intervals. The solid line shows an unadjusted sinusoidal regression model fit to the data. Right upper panel: seasonal variation of copeptin in individuals with BMI <25 kg/m^2^ presented as adjusted mean log copeptin z-score by month (solid line) with 95% confidence intervals (grey area). The dotted line shows seasonal variation of copeptin as adjusted median copeptin. Plotting of models is based on average age and proportions of categories of sex, physical activity, and cohort. Left lower panel: seasonal variation of copeptin in individuals with BMI ≥25 kg/m^2^ similarly presented as that for those with BMI <25 kg/m^2^ in the left upper panel. Right lower panel: seasonal variation of copeptin in individuals with BMI ≥25 kg/m^2^ similarly presented as that for those with <25 kg/m^2^ in the right upper panel.

Individuals with BMI <25 kg/m^2^ had lower predicted median copeptin (5.45 pmol/L [95% CI 5.38; 5.51]) compared to individuals with BMI ≥25 kg/m^2^ (6.02 pmol/L [95% CI 5.96; 6.09]). However, individuals with BMI <25 kg/m^2^ had a higher adjusted relative seasonal variation compared to individuals with BMI ≥25 kg/m^2^ (difference in log copeptin z-score 0.10 [95% CI 0.028; 0.17]) but similar adjusted absolute seasonal variation (0.68 pmol/L [95% CI 0.50; 0.85] vs. 0.60 pmol/L [95% CI 0.41; 0.80]). As outlined in Fig. 4 and Table S5, there was no difference in the timing of peak or nadir in the seasonal variation of copeptin by BMI.

### The seasonal variation in copeptin related to the risk of incident diabetes and CAD

Among individuals for whom we had access to incident diabetes diagnoses during follow-up (*n* = 13,572, *n* events = 1091, median follow-up time 5.6 years, total follow-up time 123,003 person-years), we found that an increase in mean (log) copeptin (z-score) corresponding to the seasonal variation (i.e., the difference between peak and nadir) was associated with an increased hazard ratio of diabetes (1.040 [95% CI 1.027; 1.053]).

Among individuals for whom we had access to incident CAD diagnoses during follow-up (*n* = 13,670, *n* events = 1103, median follow-up time 11.1 years, total follow-up time 131,086 person-years), we found that an increase in mean (log) copeptin (z-score) corresponding to the seasonal variation (i.e., the difference between peak and nadir) was associated with an increased hazard ratio of CAD (1.020 [95% CI 1.008; 1.033]).

### Additional results

Compared to the main analysis, the seasonal variation of copeptin showed a similar pattern in the subgroup analyses either adjusted for serum creatinine, smoking, or restricted to individuals without prevalent diabetes or CAD disease.

## Discussion

Our key finding is that VP concentration, estimated through the surrogate marker copeptin, exhibits a distinct seasonal rhythm in adults living in a temperate climate, with the peak occurring during winter and nadir in late summer. This seasonal variation is evident in both men and women, among both older and younger individuals, among both normal weight and overweight individuals, and in all five cohorts analyzed. The magnitude of the seasonal variation in VP is also large enough to correspond to relevant risk increases of incident diabetes and CAD at the population level.

The seasonal variation of VP has been largely unexplored. Two small previous studies investigated seasonal variation of VP in young, healthy volunteers in a temperate climate (Japan and Austria) [13, 22]. In contrast to our current study, these studies found a higher VP in the warm season than during the cold season. To explain these seemingly incongruent observations, we propose as a cohesive hypothesis that there is a nonlinear effect of outdoor temperature on VP concentration. In other words, we suggest that the relationship between temperature and VP is J-shaped with higher VP concentrations both at relatively low and high temperatures. This tentative explanatory model is supported by the results of a large study in adults from South Korea, in which the authors found several complementary markers of dehydration (however, not copeptin) to be slightly increased in both relatively low and high temperatures [23]. Thus, a small decrease in relative hydration during lower temperatures in winter appears to be concurrent with a more substantial increase of VP concentration.

The underlying mechanisms behind the "winter cardiovascular disease phenomenon" of increased cardiovascular disease incidence and mortality [24] are unknown [5]. We hypothesize that this phenomenon is partly attributable to seasonal variation in VP secretion. We and others have identified VP as a causal candidate of metabolic and cardiovascular disease [25, 26], and human and animal experiments have shown beneficial metabolic effects from water-induced VP and copeptin reduction [10, 17].

The physiological effects of VP are mediated through three receptors. The antidiuretic action of VP mainly depends on the V2 receptor (V2R) in the renal collecting duct [27]. The metabolic effects of VP are mediated via the V1a receptor (V1aR) and V1b receptor (V1bR) and include gluconeogenesis and glycogenolysis [28, 29], glucagon secretion [30], antilipolysis and hepatic production of triglycerides [31, 32], and adrenocorticotropic hormone (ACTH) release and hypercortisolism [33, 34]. Increased V1aR and V1bR activity would thus be expected to result in a disadvantageous glucometabolic profile. Furthermore, in the vessels, the V1aR mediates platelet aggregation and vasoconstriction [35, 36]. Several cardiovascular risk factors, including blood pressure, lipids, and glucose [8, 37], exhibit to a varying degree a seasonal pattern with a peak during winter. Given the physiological effects of VP, increased concentration during winter is concordant with previous observations of an increase in urine concentration [38], elevated plasma glucose and triglycerides [8, 37], elevated blood pressure [8], increased hemostasis [9], and elevated cortisol [2] during winter. Lowered VP secretion in winter might thus affect several of these pathways and potentially result in a relevant reduction of incident cardiometabolic disease.

Low water intake is the key determinant of high plasma VP and copeptin during normal conditions [16, 39]. Consequently, the easiest way to lower VP and copeptin concentration in plasma is to drink water [16, 39]. Previous epidemiological studies have found increased water intake during the warm season [40, 41]. Another study found serum osmolality among elderly to be higher during the spring, and urine to be more concentrated during the winter and the spring, than during the summer and autumn [38], further pointing at a less hydrated state, and thus elevated VP concentration, during the cold season.

Thirst, hydration status, and drinking behavior are complex entities and decreased thirst, as well as increased diuresis, during the cold season may be linked to other environmental stimuli, such as daylight or temperature. Experiments in rats suggest that cold-induced diuresis might be caused by downregulation of V2R in the kidney, which in turn results in increased urine output [42]. These results suggest that cold-induced diuresis is due to suppression of renal V2R rather than inhibition of VP secretion. Experimental evidence suggests that low outdoor temperature decreases thirst [43]. The underlying mechanism may be an effect of vasoconstriction in peripheral tissues, resulting in increased volume in internal vascular beds mimicking volume expansion, which in turn affects VP secretion and thirst [44] but may also be linked to the fact that cold water is a better thirst quencher than room-temperature water [45]. A link between water intake, VP, and circadian rhythmicity has been established in rodents in a study showing anticipatory thirst before sleep independent of current serum osmolality. The increased water intake is shown to be a consequence of increased VP signaling (as a neurotransmitter) in the hypothalamic suprachiasmatic nucleus [46]. Our results, showing a peak in copeptin concentration during the cold season, may thus be a consequence of decreased water intake, which in turn may be a result of environmental stimuli such as low outdoor temperature, as discussed above, or decreased amount of daylight.

Considering daylight more specifically, we further hypothesize that the winter peak of VP concentration is partly linked to an increase in melatonin secretion due to decreased daylight. Melatonin is released at night to synchronize the circadian rhythm and increases circulating concentrations of VP in humans [47]. A previous study from Southern Sweden has shown that melatonin concentration increases during winter [48]. Moreover, a study on participants in the EpiHealth cohort found short sleep duration (<6 h) to be less frequent in winter compared to summer [49]. Even though no overall seasonal pattern of sleep length or sleep-related problems mirroring that of VP concentration was observed across the four seasons, one may speculate that longer sleep duration due to longer nights could increase time without hydration during winter, thereby leading to variation in VP concentration by season.

Copeptin also increases with psychological and physiological stress [11, 50], and VP production in the human brain seems to play an important role in forming circadian rhythmicity and sleeping patterns [51]. In addition, a dysregulated VP system has been proposed as an underlying etiological mechanism of depression [52], copeptin has been suggested as a marker of treatment response to antidepressants [53], and there is a seasonal variation of affective disorders. All in all, the extent to which the observed seasonality of VP concentration is relevant for stress and sleep is unknown and warrants further investigation.

The 1-month-earlier peak and nadir of copeptin in women may hypothetically be related to a difference in responsiveness to these environmental stimuli between sexes. Differences between men and women in the timing of peak and nadir have previously been demonstrated in other hormonal systems [54].

If increased VP concentration is causally linked to increased cardiovascular risk in the winter, the most convenient and safe way to lower this risk would be to drink more water in the winter. Copeptin concentration in plasma is lowered by approximately 40% after an acute water load of 1 L and remains low for 4 h [16]. In this study, we found the seasonal variation in median copeptin to be approximately 10%. Furthermore, we have previously found that the low-drinking part of the population (low 24-h urine volume but high urine osmolality and plasma copeptin) has the largest water-induced copeptin reduction [10].

We found that the seasonal variation in copeptin corresponded to a 4% increased risk of diabetes and a 2% increased risk of CAD. We suggest that a lifestyle intervention of moderately increased water intake during the cold season would produce great overall benefit if a large segment of the population was included, given that a causal link between VP and cardiometabolic status is established. We previously found approximately half of adults to have a daily total water intake below the adequate daily intake of 2.5 L for men and 2L for women [21, 55]. Vitamin D deficiency has previously been widely hypothesized to be important for the seasonal variation of cardiovascular disease but must be regarded as an unlikely etiological factor given the sizable evidence of no cardiovascular protection by vitamin D supplementation [56].

We acknowledge that the study has limitations. First, two different copeptin analysis methods were used. However, as these two methods were recently found to yield comparable copeptin concentrations [20], it should not bias our results by season, and our observations are largely similar in all five cohorts. Second, we have analyzed individual-level data from five different cohorts. Four out of five studies have been run from the same clinical research unit in Malmö, but we acknowledge minor differences in the collection of the questionnaire-based physical activity data. Finally, it is known that individuals with diabetes and elevated cardiovascular risk have elevated copeptin concentration. Thus, we cannot rule out that the observed seasonality of copeptin during the cold season is partly driven by a more dysregulated metabolic profile.

In conclusion, the seasonal variation of the VP marker copeptin corresponds to a relevant increase of disease risk at the population-level and mirrors the known variation in cardiometabolic status and incident CAD across the year. Moderately increased water intake might mitigate the winter peak of cardiometabolic disease. Our results challenge the paradigm that VP concentration peaks in the summer.

## Supplementary Material

Supplementary material

## Figures and Tables

**Table 1:** Sample description with baseline characteristics of the five pooled cohorts (n = 25,907)

Age, years	59.6 (10.4)
Men, *n* (%)	12,852 (49.6)
Body mass index, kg/m^2^	26.5 (4.27)
Physical activity^a^, *n* (%)	
Sedentary lifestyle	3304 (12.8)
Low-grade exercise	11,322 (43.7)
Regular exercise	7412 (28.6)
Regular intense exercise	3869 (14.9)
Plasma copeptin^b^, pmol/ L	5.46 (3.63; 8.66)

*Note:* Values are given as mean (standard deviation) if nothing else is specified.

a During leisure time.

b Median (25th percentile; 75th percentile)
