# Supplementary material for: Seasonal variation of vasopressin and its relevance for the winter peak of cardiometabolic disease: A pooled analysis of five cohorts
Source: J Intern Med. 2022 Apr 17;292(2):365–76. doi: 10.1111/joim.13489 (PMC7613412; doi:10.1111/joim.13489)
Supplement: Supplementary file 1 — Figure S1: Mean temperature and daylight (January‐December) in Malmö, Sweden. Figure S2: Seasonal variation of vasopressin estimated through copeptin in the Malmö Diet and Cancer Cardiovascular cohort. Figure S3: Seasonal variation of vasopressin estimated through copeptin in the Malmö Preventive Project cohort. Figure S4: Seasonal variation of vasopressin estimated through copeptin in the EpiHealth cohort. Figure S5: Seasonal variation of vasopressin estimated through copeptin in the Swedish CArdioPulmonary BioImage Study cohort. Figure S6: Seasonal variation of vasopressin estimated through copeptin in the Malmö Offspring Study cohort. Table S1: Sample description by cohort. Table S2: Seasonal variation, peak and nadir of copeptin. Table S3: Seasonal variation, peak and nadir of copeptin by sex. Table S4: Seasonal variation, peak and nadir of copeptin by age. Table S5: Seasonal variation, peak and nadir of copeptin by BMI. [file JOIM-292-365-s001.docx]

**Supplemental Material**

**Seasonal variation of vasopressin and its relevance for the winter peak of cardiometabolic disease: a pooled analysis of five cohorts**

Sofia Enhörning, MD, PhD; Olle Melander, MD, PhD; Gunnar Engström, MD, PhD; Sölve Elmståhl, MD, PhD Lars Lind, MD, PhD; Peter M. Nilsson, MD, PhD; Mats Pihlsgård, PhD; Simon Timpka, MD, PhD

**List of contents**

1. **Supplemental Methods**
2. **Supplemental Results**

*List of supplemental tables*

eTable 1. Sample description by cohort

eTable 2. Seasonal variation, peak and nadir of copeptin

eTable 3. Seasonal variation, peak and nadir of copeptin by sex

eTable 4. Seasonal variation, peak and nadir of copeptin by age

eTable 5. Seasonal variation, peak and nadir of copeptin by BMI

*List of supplemental figures*

eFigure 1. Mean temperature and daylight (January-December) in Malmö, Sweden

eFigure 2. Seasonal variation of vasopressin estimated through copeptin in the Malmö Diet and Cancer Cardiovascular cohort

eFigure 3. Seasonal variation of vasopressin estimated through copeptin in the Malmö Preventive Project cohort

eFigure 4. Seasonal variation of vasopressin estimated through copeptin in the EpiHealth cohort

eFigure 5. Seasonal variation of vasopressin estimated through copeptin in the Swedish CArdioPulmonary BioImage Study cohort

eFigure 6. Seasonal variation of vasopressin estimated through copeptin in the Malmö Offspring Study cohort

1. **Supplemental References**
2. **Supplemental Methods**

*Malmö Diet and Cancer Cardiovascular Cohort*

The Malmö Diet and Cancer study (MDC) is a Swedish population-based prospective cohort consisting of 30,447 individuals surveyed in 1991 to 1996 with the aim of exploring the links between dietary patterns and cancer [1]. Adults age 45 to 69 years living in Malmö, Sweden, were eligible for the study. Between October 1991 and February 1994, a random 50% were invited to also take part in a sub-study of the epidemiology of carotid artery disease [2]. This sample is referred to as the cardiovascular cohort (MDC-CC) and consists of 6,103 individuals. Of those, 5,540 accepted an invitation for blood sampling under standardized fasting circumstances. Plasma samples were frozen to −80° C for later analyses.

Furthermore, participants underwent a physical examination and laboratory assessment. Cigarette smoking and leisure time physical activity was assessed by a self-administered questionnaire. Leisure time physical activity was assessed on the basis of a list of activities adapted from the Minnesota Leisure Time Physical Activity instrument [3]. A score was obtained by multiplying the minutes per week spent on a specific activity by an activity-specific factor. Based on this score, the population was ranked into quartiles. A sedentary lifestyle was defined as the lowest quartile, whereas regular exercise was defined as the highest quartile.

In this cohort, 14 men and 75 women had a copeptin value below the reported LLD (0.4 pmol/L). In these participants (89 of the total n), we set copeptin to the LLD to be conservative.

*Malmö Preventive Project*

The study population of this study was a subpart of the Malmö Preventive Project (MPP), a Swedish single-center population-based prospective cohort previously described in detail [4]. MPP started in 1974 with the aim to examine a significant part of the adult population living in Malmö, i.e. the largest city of southern Sweden, in order to find high-risk individuals for preventive intervention on cardiovascular risk factors, alcohol abuse, impaired glucose tolerance, and breast cancer. Between 1974 and 1992, approximately 33,000 individuals, out of which around 2/3 were men, attended the screening program (71% participation rate). The large majority of the participants were born in Sweden and of Caucasian ethnicity. Between 2002 and 2006, all subjects who were alive and still residing in Malmö were invited for a re-examination which is previously described in detail and in which 18,238 individuals participated (63% men, 72% participation rate) [5]. The participants were at this point between 53-81 years old. At the re-examination, participants underwent a medical history, physical examination and laboratory assessment. Cigarette smoking was assessed by a self-administered questionnaire, with current cigarette smoking deﬁned as any use within the past year. Leisure time physical activity was estimated by the Saltin-Grimby physical activity scale, a 4-grade scale of physical activity level during leisure time during the past 12 months ranging from a sedentary lifestyle to regular exercise [6].

Fasting plasma samples were frozen to −80° C for later analyses. The plasma samples selected for analysis of copeptin were randomly chosen from all samples collected during the re-examination, with the only exclusion criterion being prior participation in the, at that time, only other large population-based prospective cohort study from Malmö, MDC-CC, resulting in non-existing overlap between the two studies.

*EpiHealth*

The primary aim of the EpiHealth cohort study is to study interactions between genes and environmental/lifestyle factors in a large cohort regarding development of common disorders such as cardiovascular diseases and degenerative disorders [7, 8]. The EpiHealth cohort study started in 2011 and is derived from the Swedish population and consists of randomly selected males and females aged 45 to 75 years. The current study used data from individuals invited between 2011 and 2016 in Malmö. The participants filled out an extensive internet-based questionnaire, in which for example cigarette smoking and leisure time physical activity was assessed. In the EpiHealth cohort, leisure time physical activity was estimated by a 7-grade scale ranging from no exercise to regular intensive training. To better harmonize with the Saltin-Grimby scale used in the other studies, the 7-grade scale was collapsed to a 4-grade scale. Fasting plasma samples (6 hours of fasting was required) were collected and frozen to −80° C for later analyses.

*The Swedish CArdioPulmonary BioImage Study*

The Swedish CArdioPulmonary BioImage Study (SCAPIS) was initiated as a major national effort in Sweden to reduce mortality and morbidity from cardiopulmonary diseases. A random selection of 30,154 men and women, aged 50–64 years and residing in and around six Swedish cities (Gothenburg, Linköping, Malmö/Lund, Stockholm, Umeå and Uppsala), were recruited between 2013 and 2018. The participants completed an extensive questionnaire including information about current cigarette smoking and leisure time physical activity. Leisure time physical activity was estimated by the Saltin-Grimby physical activity scale, a 4-grade scale of physical activity level during leisure time during the past 12 months ranging from a sedentary lifestyle to regular exercise [6]. The participants underwent a health examination including anthropometry, blood sampling and different types of imaging. The overall participation rate was 50%. The current study used data from individuals residing in the geographical area of Malmö/Lund [9].

*The Malmö Offspring Study*

The single-center ongoing Malmö Offspring Study (MOS) started in 2013 with the main aim to map family traits of chronic diseases across three generations [10]. Children and grandchildren of participants in the Malmö Diet Cancer Study (see above) are recruited. Thus, the inclusion criterion for MOS is to have a parent or grandparent in the Malmö Diet and Cancer Cohort. So far, around 5000 participants between 18 and 71 years have been included in the study. At a clinic visit after an overnight fast, the participants undergo a health examination including anthropometry, blood sampling and complete extensive questionnaires (including self-reported physical activity). Leisure time physical activity is estimated by the Saltin-Grimby physical activity scale, a 4-grade scale of physical activity level during leisure time during the past 12 months ranging from a sedentary lifestyle to regular exercise [6]. Plasma samples are frozen to −80° C for later analyses. In May 2017, the MOS study had reach halftime according to initial recruitment goals. In an interim analysis from that time point the participation rate was 47%. The current study used data from the study halftime.

*Ascertainment of diagnosis of diabetes and coronary artery disease*

In the MDC-CC, the MPP and the Epi-Health cohorts, diagnoses were identified by linking a 10-digit personal identification number of each Swedish citizen with different registers. Prevalent diabetes was defined as a registration of a diabetes diagnosis before or at the day of blood sampling, and incident diabetes was defined as registration of a diabetes diagnosis after the day of blood sampling. Prevalent and incident diabetes diagnoses were captured in either the Swedish National Patient Register, which is a principal source of data for numerous research projects and covers more than 99% of all somatic and psychiatric hospital discharges and Swedish hospital-based outpatient care [11], or in the Swedish Cause-of-Death Register, which comprises all deaths among Swedish residents occurring in Sweden or abroad [12], if the cause of death was diabetes, or if a prescription of antidiabetic medication was registered in the Swedish Prescribed Drug Register [13]. In the MDC-CC and the MPP cohort, diabetes diagnoses could also be captured in the nationwide Swedish National Diabetes Register [14] or in the regional Diabetes 2000 register of the Scania region, of which Malmö is the largest city [15], or if an individual had two HbA1c recordings ≥6.0% using the Swedish Mono-S standardization system (corresponding to 7.0% according to the US National Glycohemoglobin Standardization Program) in the Malmö HbA1c register, which analyzed and catalogued all HbA1c samples at the Department of Clinical Chemistry taken in institutional and noninstitutional care in the greater Malmö area from 1988 onward.

Prevalent CAD was defined as a registration of CAD before or at the day of blood sampling, and incident CAD was defined as registration of CAD after the day of blood sampling. Prevalent and incident CAD diagnoses were captured in either the Swedish National Patient Register [11], the Swedish Cause-of-Death Register [12] or the Swedish Coronary Angiography and Angioplasty Registry (SCAAR) [16]. CAD was defined as coronary revascularization (coronary artery bypass surgery or percutaneous intervention), fatal or non-fatal myocardial infarction, or death due to ischemic heart disease.

1. **Supplemental Results**

| **eTable 1.** Sample description by cohort | | | | | |
| --- | --- | --- | --- | --- | --- |
|  | **Malmö Diet and Cancer Cardiovascular cohort** | **Malmö Preventive Project cohort** | **EpiHealth cohort** | **Swedish CArdioPulmonary BioImage Study cohort** | **Malmö Offspring Study cohort** |
| N | 5,028 | 5,338 | 8,013 | 5,448 | 2,080 |
| Age, years | 57.5 (6.0) | 69.4 (6.2) | 60.8 (8.4) | 57.5 (4.3) | 40.0 (14.1) |
| Men, n (%) | 2,041 (40.6) | 3,727 (69.8) | 3,547 (44.3) | 2,548 (46.8) | 989 (47.6) |
| Body mass index, kg/m^2^ | 25.7 (3.93) | 27.2 (4.22) | 26.3 (4.08) | 27.2 (4.53) | 25.8 (4.65) |
| Physical activity,^a^ n (%) |  |  |  |  |  |
| Sedentary lifestyle | 1,186 (23.6) | 641 (12.0) | 518 (6.5) | 801 (14.7) | 158 (7.6) |
| Low-grade exercise | 1,249 (24.8) | 3,827 (71.7) | 2,822 (35.2) | 2,675 (49.1) | 749 (36.0) |
| Regular exercise | 1,321 (26.3) | 863 (16.2) | 3,274 (40.9) | 1,399 (25.7) | 555 (26.7) |
| Regular intense exercise | 1,272 (25.3) | 7 (0.1) | 1,399 (17.5) | 573 (10.5) | 618 (29.7) |
| Plasma copeptin, ^b^ pmol/l | 5.15 (3.20; 8.18) | 7.15 (4.31; 11.94) | 4.99 (3.57; 7.78) | 5.14 (3.60; 7.96) | 5.67 (3.76; 8.52) |
| Values are given as mean (standard deviation) if nothing else specified  ^a^ During leisure time  ^b^ Median (25th percentile; 75th percentile) | | | | | |

| **eTable 2.** Seasonal variation, peak and nadir of copeptin | | | | | | |
| --- | --- | --- | --- | --- | --- | --- |
|  | **2 x Amplitude** | | **Peak** | | **Nadir** | |
|  | Estimate | 95% CI | Estimate | 95% CI | Estimate | 95% CI |
| Z-score log copeptin unadjusted | 0.23 | 0.20; 0.27 | 27FEB | 17FEB; 08MAR | 28AUG | 19AUG; 07SEP |
| Z-score log copeptin adjusted | 0.20 | 0.17; 0.24 | 26FEB | 16FEB; 09MAR | 28AUG | 17AUG; 07SEP |
| Median copeptin adjusted | 0.62  (pmol/L) | 0.50; 0.74 (pmol/L) | 02MAR | 18FEB; 14MAR | 31AUG | 20AUG; 12SEP |
| CI: Confidence Interval | | | | | | |

| **eTable 3.** Seasonal variation, peak and nadir of copeptin by sex | | | | | | |
| --- | --- | --- | --- | --- | --- | --- |
|  | **2 x Amplitude** | | **Peak** | | **Nadir** | |
|  | Estimate | 95% CI | Estimate | 95% CI | Estimate | 95% CI |
| **Men** | | | | | | |
| Z-score log copeptin unadjusted | 0.23 | 0.18; 0.28 | 16MAR | 02MAR; 30MAR | 15SEP | 31AUG; 29SEP |
| Z-score log copeptin adjusted | 0.18 | 0.14; 0.23 | 18MAR | 01MAR; 04APR | 16SEP | 30AUG; 04OCT |
| Median copeptin adjusted | 0.98 (pmol/L) | 0.73; 1.23 (pmol/L) | 13MAR | 25FEB; 29MAR | 12SEP | 27AUG; 28SEP |
| **Women** | | | | | | |
| Z-score log copeptin unadjusted | 0.25 | 0.20; 0.30 | 13FEB | 01FEB; 24FEB | 14AUG | 03AUG; 26AUG |
| Z-score log copeptin adjusted | 0.24 | 0.19; 0.29 | 12FEB | 31JAN; 24FEB | 14AUG | 01AUG; 26AUG |
| Median copeptin adjusted | 0.46 (pmol/L) | 0.33; 0.59 (pmol/L) | 18FEB | 01FEB; 07MAR | 20AUG | 03AUG; 05SEP |
| CI: Confidence Interval | | | | | | |

| **eTable 4.** Seasonal variation, peak and nadir of copeptin by age | | | | | | |
| --- | --- | --- | --- | --- | --- | --- |
|  | **2 x Amplitude** | | **Peak** | | **Nadir** | |
|  | Estimate | 95% CI | Estimate | 95% CI | Estimate | 95% CI |
| **Age ≤ 60 years** | | | | | | |
| Z-score log copeptin unadjusted | 0.21 | 0.16; 0.26 | 26FEB | 10FEB; 14MAR | 27AUG | 11AUG; 12SEP |
| Z-score log copeptin adjusted | 0.21 | 0.16; 0.26 | 27FEB | 11FEB; 15MAR | 29AUG | 13AUG; 13SEP |
| Median copeptin adjusted | 0.67 (pmol/L) | 0.51; 0.83 (pmol/L) | 19FEB | 04FEB; 06MAR | 21AUG | 06AUG; 05SEP |
| **Age > 60 years** | | | | | | |
| Z-score log copeptin unadjusted | 0.19 | 0.14; 0.24 | 06MAR | 17FEB; 23MAR | 05SEP | 18AUG; 22SEP |
| Z-score log copeptin adjusted | 0.18 | 0.13; 0.23 | 09MAR | 19FEB; 27MAR | 08SEP | 21AUG; 26SEP |
| Median copeptin adjusted | 0.68 (pmol/L) | 0.48; 0.89 (pmol/L) | 12MAR | 21FEB; 01APR | 11SEP | 23AUG; 30SEP |
| CI: Confidence Interval | | | | | | |

| **eTable 5.** Seasonal variation, peak and nadir of copeptin by BMI | | | | | | |
| --- | --- | --- | --- | --- | --- | --- |
|  | **2 x Amplitude** | | **Peak** | | **Nadir** | |
|  | Estimate | 95% CI | Estimate | 95% CI | Estimate | 95% CI |
| **BMI < 25 kg/m^2^** | | | | | | |
| Z-score log copeptin unadjusted | 0.28 | 0.23; 0.34 | 28FEB | 16FEB; 12MAR | 29AUG | 17AUG; 11SEP |
| Z-score log copeptin adjusted | 0.26 | 0.21; 0.32 | 27FEB | 14FEB; 12MAR | 28AUG | 15AUG; 10SEP |
| Median copeptin adjusted | 0.68 (pmol/L) | 0.50; 0.85 (pmol/L) | 07MAR | 19FEB; 22MAR | 05SEP | 21AUG; 21SEP |
| **BMI ≥ 25 kg/m^2^** | | | | | | |
| Z-score log copeptin unadjusted | 0.19 | 0.15; 0.24 | 27FEB | 12FEB; 13MAR | 28AUG | 14AUG; 11SEP |
| Z-score log copeptin adjusted | 0.17 | 0.12; 0.21 | 24FEB | 08FEB; 13MAR | 26AUG | 09AUG; 11SEP |
| Median copeptin adjusted | 0.60 (pmol/L) | 0.41; 0.80 (pmol/L) | 27FEB | 10FEB; 16MAR | 28AUG | 11AUG; 14SEP |
| CI: Confidence Interval; BMI: Body Mass Index | | | | | | |

| **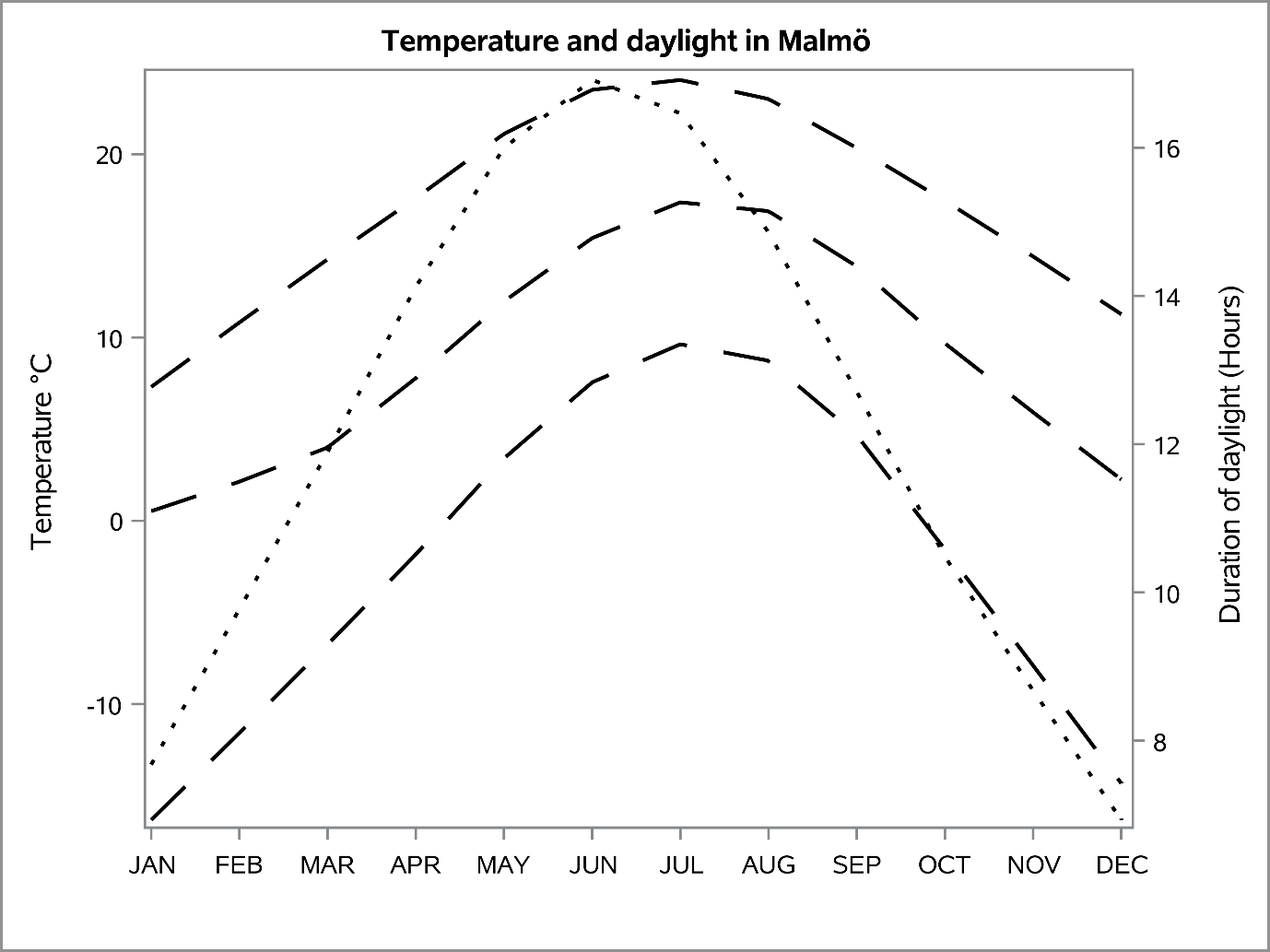** |
| --- |
| **eFigure 1. Mean temperature and daylight (January-December) in Malmö, Sweden**  On the left axis presented as dashed lines are the maximum mean per day, mean by month, and minimum mean per day temperature during January to December. On the right axis presented as dotted lines is the duration of daylight during January to December. Data on mean temperature and daylight were obtained from the Swedish Meteorological and Hydrological Institute ([http://opendata-download-metobs.smhi.se/explore/?parameter=3#](http://opendata-download-metobs.smhi.se/explore/?parameter=3)) |

| 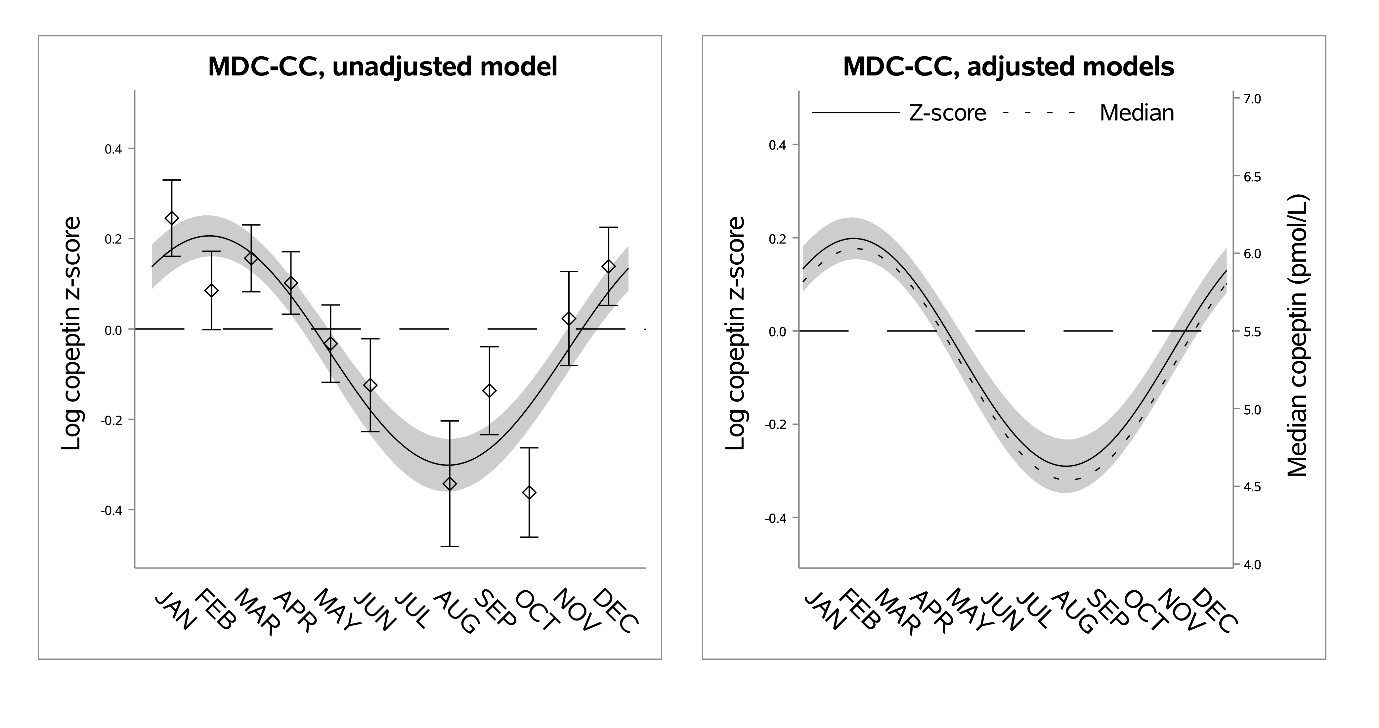 |
| --- |
| **eFigure 2. Seasonal variation of vasopressin estimated through copeptin in the Malmö Diet and Cancer Cardiovascular cohort**  Left panel: Seasonal variation of copeptin presented as mean log copeptin z-score (standardized by sex) by month with 95% confidence intervals. The solid line shows an unadjusted sinusoidal regression model fit to the data.  Right panel: Seasonal variation of copeptin presented as adjusted mean log copeptin z-score by month (solid line) with 95% confidence intervals (grey area). The dotted line shows seasonal variation of copeptin as adjusted median copeptin. Plotting of models are based on cohort average of age and BMI and cohort-specific proportions for each category of sex and physical activity.  MDC-CC: Malmö Diet and Cancer Cardiovascular cohort |

| 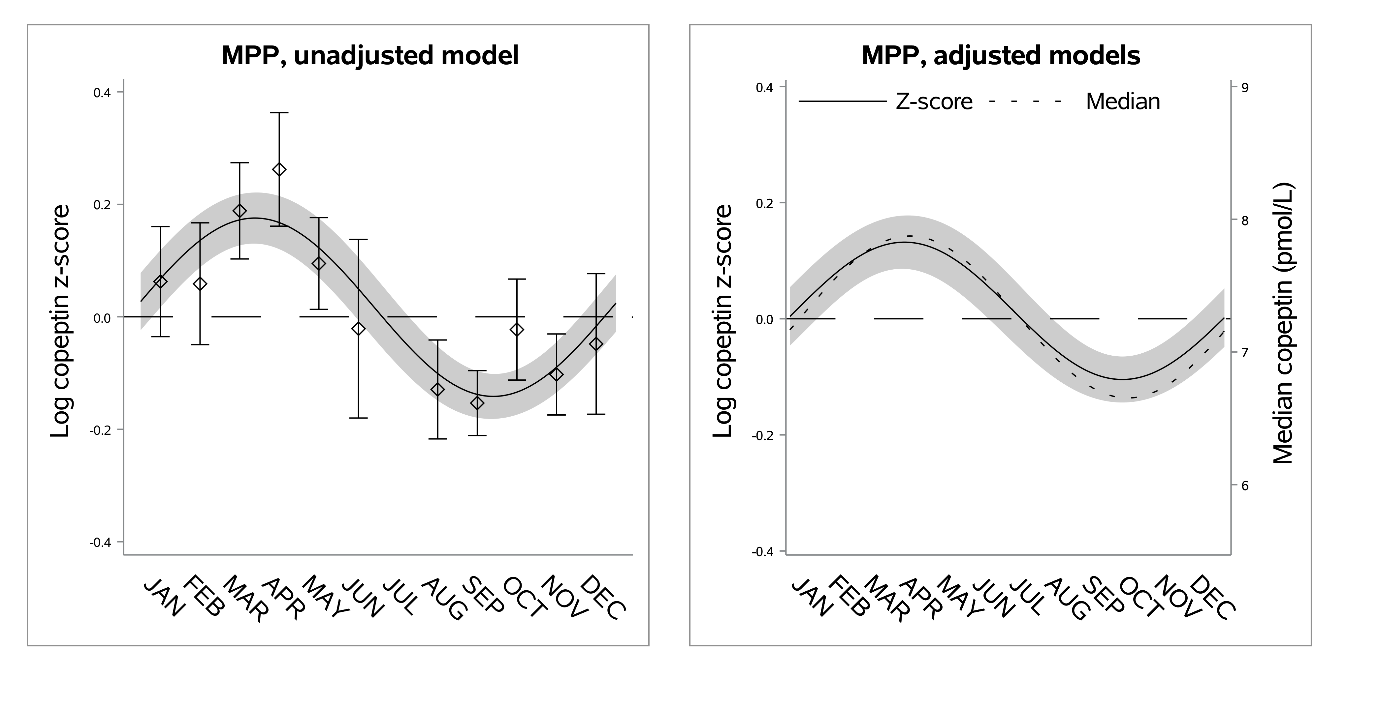 |
| --- |
| **eFigure 3. Seasonal variation of vasopressin estimated through copeptin in the Malmö Preventive Project cohort**  Left panel: Seasonal variation of copeptin presented as mean log copeptin z-score (standardized by sex) by month with 95% confidence intervals. The solid line shows an unadjusted sinusoidal regression model fit to the data.  Right panel: Seasonal variation of copeptin presented as adjusted mean log copeptin z-score by month (solid line) with 95% confidence intervals (grey area). The dotted line shows seasonal variation of copeptin as adjusted median copeptin. Plotting of models are based on cohort average of age and BMI and cohort-specific proportions for each category of sex and physical activity.  MPP: Malmö Preventive Project cohort |

| 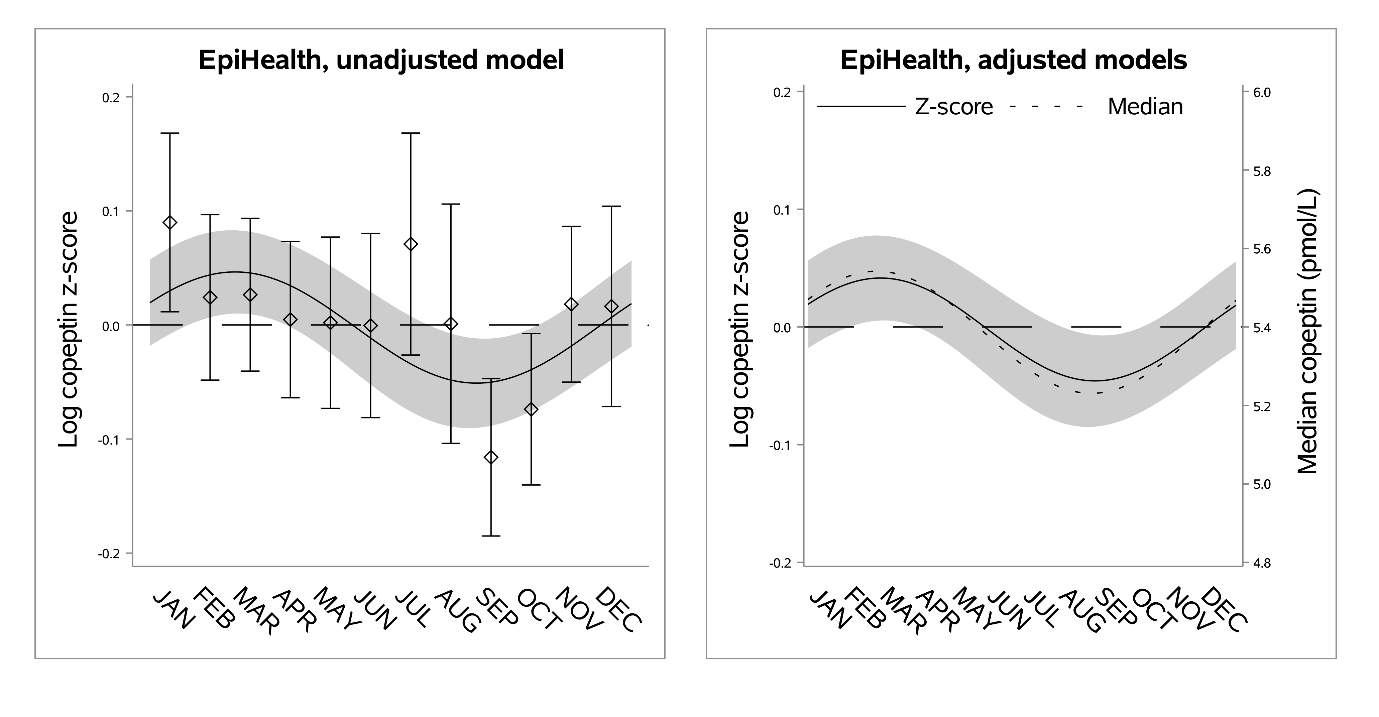 |
| --- |
| **eFigure 4. Seasonal variation of vasopressin estimated through copeptin in the EpiHealth cohort**  Left panel: Seasonal variation of copeptin presented as mean log copeptin z-score (standardized by sex) by month with 95% confidence intervals. The solid line shows an unadjusted sinusoidal regression model fit to the data.  Right panel: Seasonal variation of copeptin presented as adjusted mean log copeptin z-score by month (solid line) with 95% confidence intervals (grey area). The dotted line shows seasonal variation of copeptin as adjusted median copeptin. Plotting of models are based on cohort average of age and BMI and cohort-specific proportions for each category of sex and physical activity.  EpiHealth: EpiHealth cohort |

| 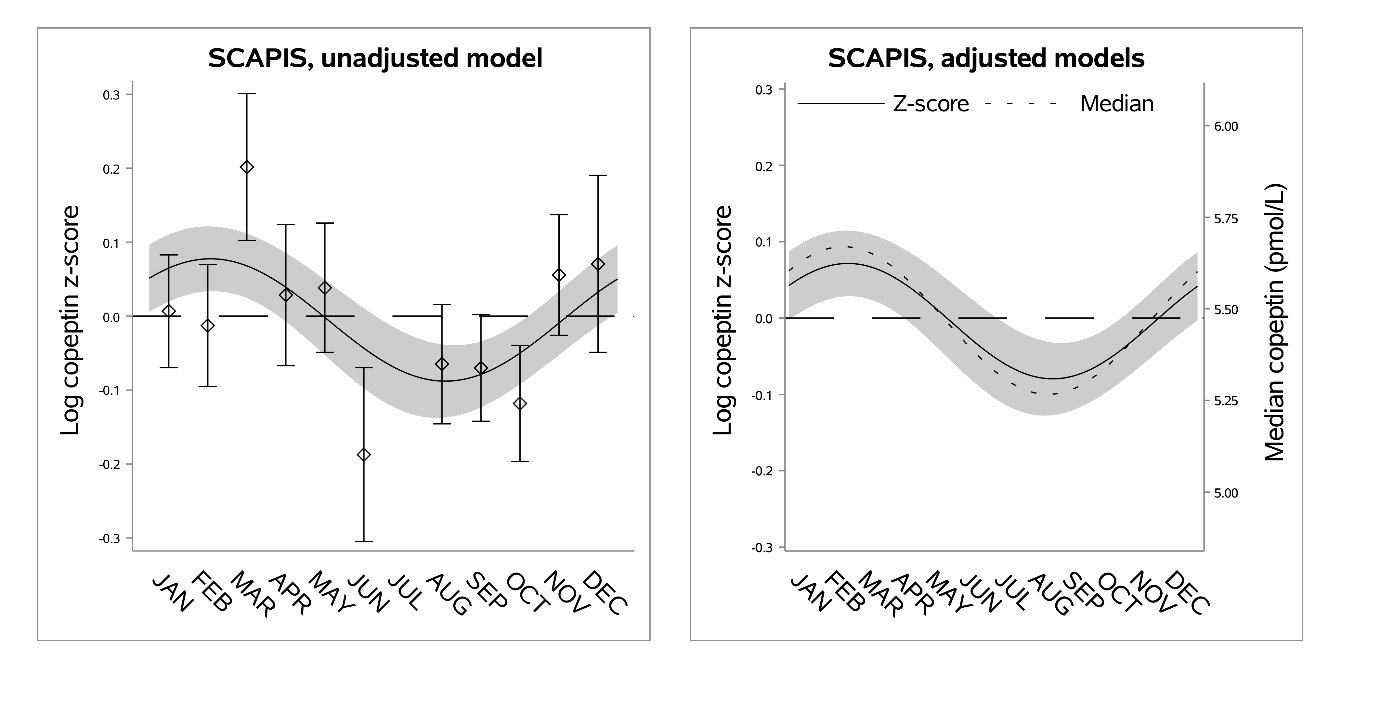 |
| --- |
| **eFigure 5. Seasonal variation of vasopressin estimated through copeptin in the Swedish CArdioPulmonary BioImage Study cohort**  Left panel: Seasonal variation of copeptin presented as mean log copeptin z-score (standardized by sex) by month with 95% confidence intervals. The solid line shows an unadjusted sinusoidal regression model fit to the data.  Right panel: Seasonal variation of copeptin presented as adjusted mean log copeptin z-score by month (solid line) with 95% confidence intervals (grey area). The dotted line shows seasonal variation of copeptin as adjusted median copeptin. Plotting of models are based on cohort average of age and BMI and cohort-specific proportions for each category of sex and physical activity.  SCAPIS: Swedish CArdioPulmonary BioImage Study cohort |

| 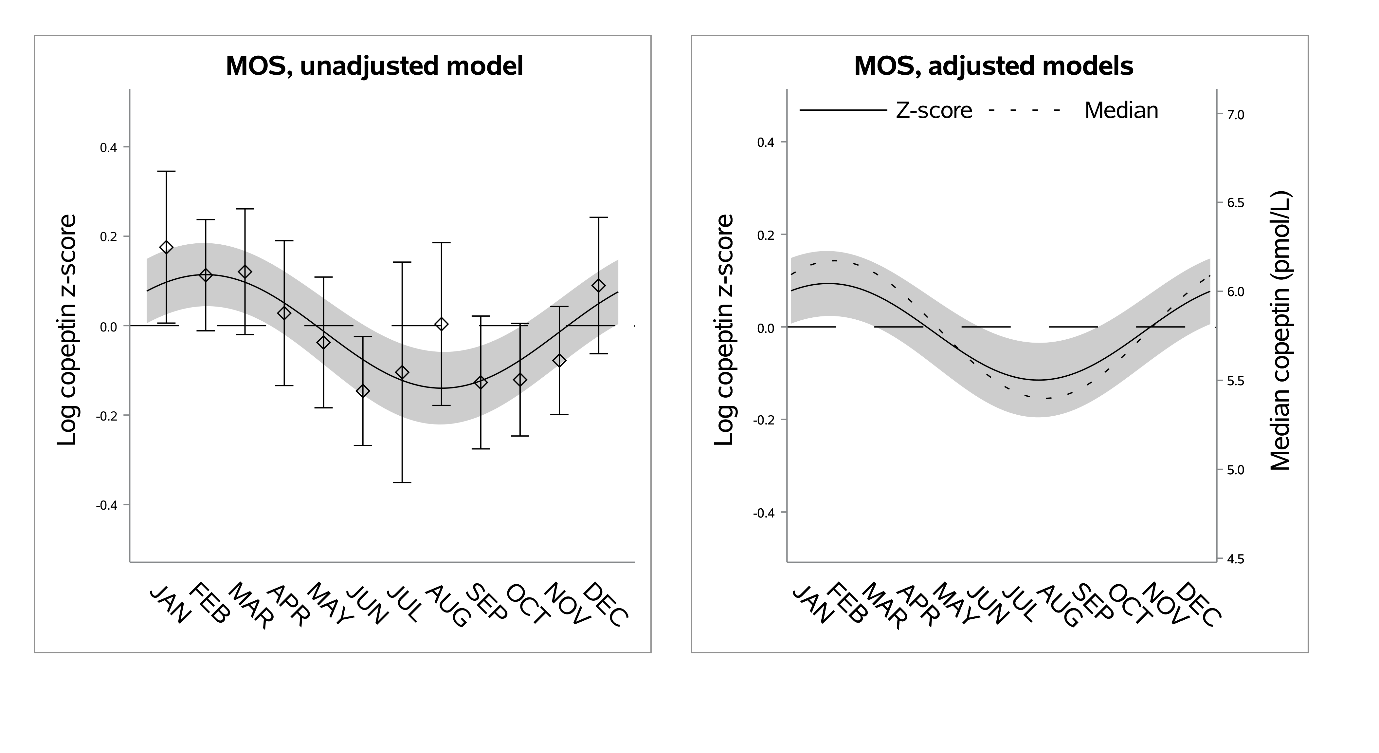 |
| --- |
| **eFigure 6. Seasonal variation of vasopressin estimated through copeptin in the Malmö Offspring Study cohort**  Left panel: Seasonal variation of copeptin presented as mean log copeptin z-score (standardized by sex) by month with 95% confidence intervals. The solid line shows an unadjusted sinusoidal regression model fit to the data.  Right panel: Seasonal variation of copeptin presented as adjusted mean log copeptin z-score by month (solid line) with 95% confidence intervals (grey area). The dotted line shows seasonal variation of copeptin as adjusted median copeptin. Plotting of models are based on cohort average of age and BMI and cohort-specific proportions for each category of sex and physical activity.  MOS: Malmö Offspring Study cohort |

**C. Supplemental References**

1. Berglund G, Elmstahl S, Janzon L, Larsson SA. The Malmo Diet and Cancer Study. Design and feasibility. *J Intern Med* 1993; **233:** 45-51.

2. Persson M, Hedblad B, Nelson JJ, Berglund G. Elevated Lp-PLA2 levels add prognostic information to the metabolic syndrome on incidence of cardiovascular events among middle-aged nondiabetic subjects. *Arterioscler Thromb Vasc Biol* 2007; **27:** 1411-1416.

3. Taylor HL, Jacobs DR, Jr., Schucker B, Knudsen J, Leon AS, Debacker G. A questionnaire for the assessment of leisure time physical activities. *J Chronic Dis* 1978; **31:** 741-755.

4. Berglund G, Nilsson P, Eriksson KF, Nilsson JA, Hedblad B, Kristenson H, et al. Long-term outcome of the Malmo preventive project: mortality and cardiovascular morbidity. *J Intern Med* 2000; **247:** 19-29.

5. Leosdottir M, Willenheimer R, Persson M, Nilsson PM. The association between glucometabolic disturbances, traditional cardiovascular risk factors and self-rated health by age and gender: a cross-sectional analysis within the Malmo Preventive Project. *Cardiovasc Diabetol* 2011; **10:** 118.

6. Grimby G, Borjesson M, Jonsdottir IH, Schnohr P, Thelle DS, Saltin B. The "Saltin-Grimby Physical Activity Level Scale" and its application to health research. *Scand J Med Sci Sports* 2015; **25** Suppl 4: 119-25.

7. Theorell-Haglow J, Lemming EW, Michaelsson K, Elmstahl S, Lind L, Lindberg E. Sleep duration is associated with healthy diet scores and meal patterns: results from the population-based EpiHealth study. *J Clin Sleep Med* 2020; **16:** 9-18.

8. Lind L, Elmstahl S, Bergman E et al. EpiHealth: a large population-based cohort study for investigation of gene-lifestyle interactions in the pathogenesis of common diseases. *Eur J Epidemiol* 2013; **28:** 189-197.

9. Bergstrom G, Berglund G, Blomberg A et al. The Swedish CArdioPulmonary BioImage Study: objectives and design. *J Intern Med* 2015; **278:** 645-659.

10. Brunkwall L, Jonsson D, Ericson U, Hellstrand S, Kennback C, Ostling G, et al. The Malmo Offspring Study (MOS): design, methods and first results. *Eur J Epidemiol* 2021; **36:** 103-116.

11. Ludvigsson JF, Andersson E, Ekbom A et al. External review and validation of the Swedish national inpatient register. *BMC Public Health* 2011; **11:** 450.

12. Johansson LA, Westerling R. Comparing Swedish hospital discharge records with death certificates: implications for mortality statistics. *Int J Epidemiol* 2000; **29:** 495-502.

13. Wettermark B, Hammar N, Fored CM et al. The new Swedish Prescribed Drug Register--opportunities for pharmacoepidemiological research and experience from the first six months. *Pharmacoepidemiol Drug Saf* 2007; **16:** 726-735.

14. Cederholm J, Eeg-Olofsson K, Eliasson B, Zethelius B, Nilsson PM, Gudbjornsdottir S. Risk prediction of cardiovascular disease in type 2 diabetes: a risk equation from the Swedish National Diabetes Register. *Diabetes Care* 2008; **31:** 2038-2043.

15. Lindholm E, Agardh E, Tuomi T, Groop L, Agardh CD. Classifying diabetes according to the new WHO clinical stages. *Eur J Epidemiol* 2001; **17:** 983-989.

16. Lagerqvist B, James SK, Stenestrand U et al. Long-term outcomes with drug-eluting stents versus bare-metal stents in Sweden. *N Engl J Med* 2007; **356:** 1009-1019.
